# State Public Health Actions to Prevent and Control Diabetes, Heart Disease, Obesity and Associated Risk Factors, and Promote School Health

**DOI:** 10.5888/pcd14.160437

**Published:** 2017-12-07

**Authors:** Barbara Z. Park, Letitia Cantrell, Holly Hunt, Rosanne P. Farris, Patricia Schumacher, Ursula E. Bauer

**Affiliations:** 1 Division of Diabetes Translation, National Center for Chronic Disease Prevention and Health Promotion, Centers for Disease Control and Prevention, Atlanta, Georgia; 2 Division for Heart Disease and Stroke Prevention, National Center for Chronic Disease Prevention and Health Promotion, Centers for Disease Control and Prevention, Atlanta, Georgia; 3 Division of Population Health, National Center for Chronic Disease Prevention and Health Promotion, Centers for Disease Control and Prevention, Atlanta, Georgia; 4 Division of Nutrition, Physical Activity, and Obesity, National Center for Chronic Disease Prevention and Health Promotion, Centers for Disease Control and Prevention, Atlanta, Georgia; 5 Division of Diabetes Translation, Centers for Disease Control and Prevention, Atlanta, Georgia; 6 National Center for Chronic Disease Prevention and Health Promotion, Centers for Disease Control and Prevention, Atlanta, Georgia

## Abstract

The National Center for Chronic Disease Prevention and Health Promotion at the Centers for Disease Control and Prevention funds a program to boost progress in reducing the prevalence and incidence of multiple chronic diseases and their associated risk factors. This article describes the program, State Public Health Actions to Prevent and Control Diabetes, Heart Disease, Obesity and Associated Risk Factors, and Promote School Health, and the program’s action model, design, and administration and management structure. This program is based on 4 domains of public health action: 1) epidemiology and surveillance, 2) environmental approaches, 3) health care system interventions, and 4) community programs linked to clinical services. The 4 domains of public health action leverage data to inform action, support healthy choices and behaviors, strengthen delivery of clinical preventive services, and help Americans better manage their health.

## Introduction

Chronic diseases, including heart disease, cancer, stroke, diabetes, and related risk factors, are among the leading causes of death and disability in the United States. In 2010, 7 of the top 10 causes of death were chronic diseases, which account for 86% of US health care costs (1). Furthermore, half of all adults have one or more chronic health conditions, and one-fourth of adults have 2 or more (2).

For 25 years, the National Center for Chronic Disease Prevention and Health Promotion (NCCDPHP) of the Centers for Disease Control and Prevention (CDC) has provided scientific leadership and technical expertise to state health and education departments to assist them in developing, implementing, and sustaining chronic disease prevention and health promotion programs. To facilitate greater progress in reducing the prevalence and incidence of multiple chronic diseases and their associated risk factors, NCCDPHP began funding programs to implement coordinated activities aligned with the 4 domains of public health action: 1) epidemiology and surveillance, 2) environmental approaches, 3) health care system interventions, and 4) community programs linked to clinical services. Together, the 4 domains provide a framework for addressing chronic conditions (eg, diabetes, hypertension) and their risk factors (eg, obesity) across multiple settings and sectors, and they allow CDC to support complementary strategies to prevent and manage the underlying risk factors for chronic diseases and to assist health care providers and individuals in self-managing multiple chronic conditions. By investing resources to implement key evidence-based strategies, NCCDPHP has sought to address multiple risk factors, conditions, and diseases simultaneously; improve program efficiency; increase program impact; and, ultimately, improve the health of communities.

To guide implementation of this new approach, the staff members of NCCDPHP programs reviewed evidence-based approaches and funding priorities across several chronic disease programs, developed a logic model of strategies and activities, and solicited partner feedback. This approach resulted in the creation of the program, State Public Health Actions to Prevent and Control Diabetes, Heart Disease, Obesity and Associated Risk Factors, and Promote School Health (State Public Health Actions), for state health departments.

Partnering with state health departments began in July 2013, and by June 2018, NCCDPHP will have partnered with 50 state health departments and the District of Columbia to address chronic diseases and other risk factors through the 4 domains. This approach leverages data to inform action, supports healthy choices and behaviors, strengthens delivery of clinical preventive services, and helps Americans better manage their health (3). The 4 domains provide focus for State Public Health Actions to address chronic disease at the individual level by promoting health care interventions and at the population level by developing policies and creating environments that promote health. We anticipate that this coordinated approach will lead to the following outcomes:

- Increased consumption of a healthy diet.
- Increased physical activity across the life span.
- Improved medication adherence for adults with high blood pressure or diabetes.
- Increased self-monitoring of high blood pressure tied to clinical support.
- Increased access to and participation in diabetes self-management programs and type 2 diabetes prevention programs.
- Increased breastfeeding.

**Figure 1:**
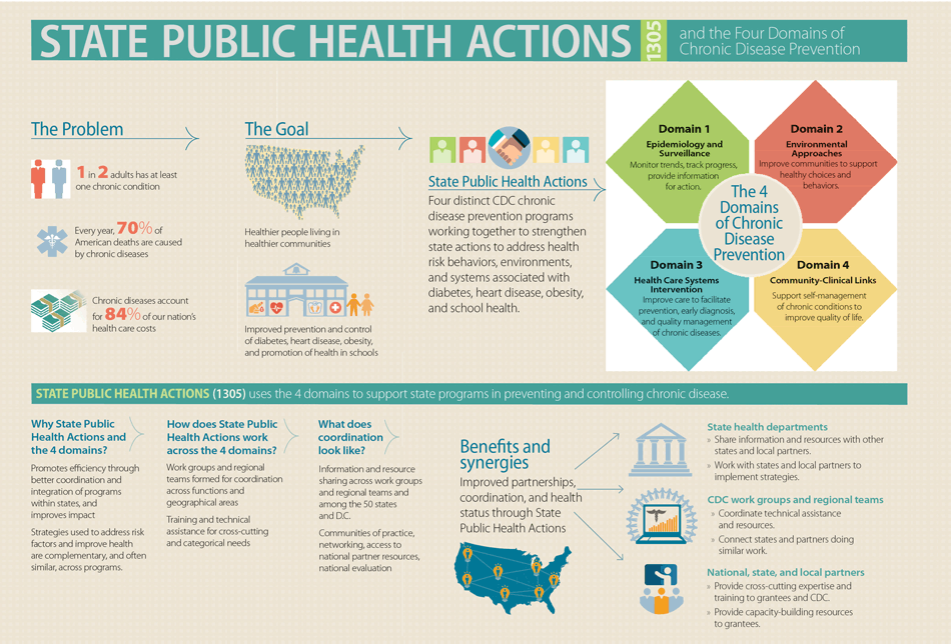
Overview of the State Public Health Actions to Prevent and Control Diabetes, Heart Disease, Obesity and Associated Risk Factors, and Promote School Health program (State Public Health Actions) for state health departments and the 4 domains of chronic disease prevention. The 4 domains provide focus for State Public Health Actions to address chronic disease at the individual level by promoting health care interventions and at the population level by developing policies and creating environments that promote health.

If successful, this approach also could lead to long-term improvement in the prevention and control of hypertension, diabetes, and obesity (Figure 1).

## Funding for State Public Health Actions

Four divisions within NCCDPHP are collaborating through a 5-year cooperative agreement to support State Public Health Actions: Diabetes Translation (http://cdc.gov/diabetes/); Heart Disease and Stroke Prevention (http://www.cdc.gov/dhdsp/); Nutrition, Physical Activity, and Obesity (http://www.cdc.gov/nccdphp/dnpao/index.html); and School Health (http://www.cdc.gov/healthyschools/) (5). The work being conducted through this cooperative agreement has basic and enhanced components. Funding for the basic component was awarded to all 50 states and the District of Columbia noncompetitively to support core public health functions such as the basic assessment strategies carried out under Domain 1 (epidemiology and surveillance). In 2013, CDC awarded approximately $28 million to support the basic component. 

**Figure 2:**
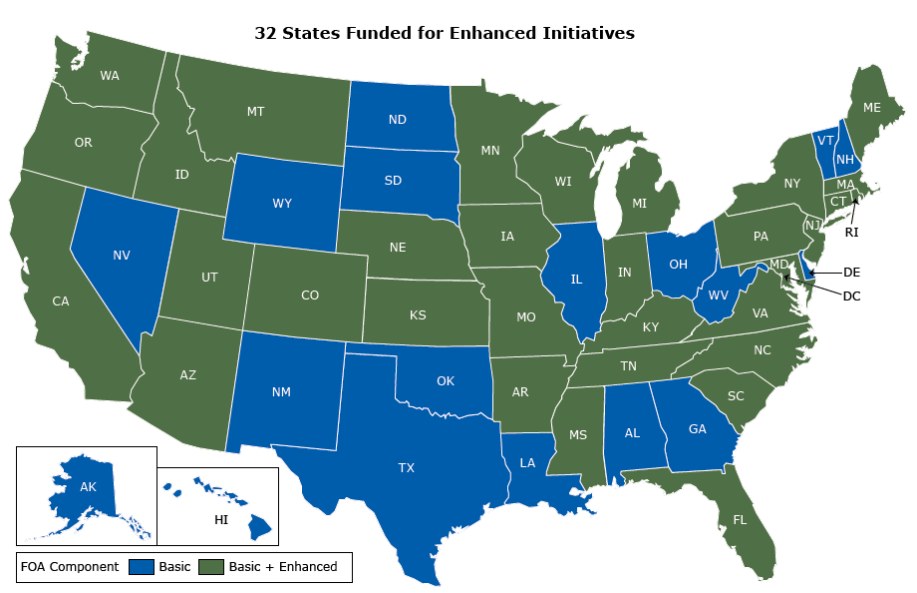
A map illustrating the level of award states received for the State Public Health Actions cooperative agreement.

Also in 2013, CDC awarded approximately $39.5 million competitively to 32 states to support enhanced strategies across Domains 2, 3, and 4 (Figure 2). The purpose of the enhanced component was to build on activities supported by basic component funding to achieve greater results. In 2014, an additional $11.9 million was awarded to the 18 states and the District of Columbia that had not received enhanced funding in 2013 to allow them to expand their efforts in diabetes, heart disease, and stroke prevention, detection, and control. The additional funding for each of these 19 grantees ranged from $336,789 to $885,199 and included state-specific adjustments for population size and poverty levels. Funding in fiscal year 2014 for the basic and enhanced components totaled approximately $79.5 million.

In 2013, CDC awarded approximately $28 million to support the basic component and $39.5 million competitively to 32 states to support enhanced strategies through the cooperative agreement. Before funding State Public Health Actions, each participating CDC program funded discrete efforts; only the Division for Heart Disease and Stroke Prevention and the Division of Diabetes Translation funded all 50 states and the District of Columbia. The State Public Health Actions effort is an attempt to purposely link strategies and activities that are mutually reinforcing to reduce duplication and maximize results (Box). For example, the Domain 3 strategy of increasing use of team-based care in health systems to improve clinical outcomes for people with hypertension and diabetes is similar to strategies already used in the health care system. Furthermore, the Domain 4 strategy of increasing use of community pharmacists to counsel individuals about how to manage their chronic conditions and adhere to medications benefits people with hypertension and diabetes. Also, greater access to, and use of, safe places to be physically active benefits the populations targeted by all 4 divisions as does increased access to healthy foods and beverages in various community settings, including schools (Domain 2). By combining forces, State Public Health Actions has resulted in an expansion of CDC’s programmatic efforts. The strategies addressed by funding to states are shown in Box.

**Box. Strategies and Activities Within State Public Health Actions**

**Basic Strategies**

Promote the adoption of food service guidelines and nutrition standards, including dietary sodium.

Promote the adoption of physical education and physical activity in schools.

Promote adoption of physical activity in early care and education and worksites.

Promote reporting of blood pressure and hemoglobin A1C control measures; as able, initiate activities that promote clinical innovations, team-based care, and self-monitoring of blood pressure to improve blood pressure control.

Promote awareness of high blood pressure among patients.

Promote awareness of prediabetes among people at high risk for type 2 diabetes.

Promote participation in diabetes self-management education programs.

**Enhanced Strategies**

Environmental approaches to promote health and support healthful behaviors

- Promote access to healthy food and beverages.
- Promote food service guidelines and nutrition standards where foods and beverages are available. Guidelines and standards should address sodium.
- Promote supportive nutrition environments in schools.
- Promote physical activity access and outreach.
- Promote physical activity in early care and education.
- Promote quality physical education and physical activity in grades kindergarten through 12 in schools.
- Promote access to breastfeeding-friendly environments.

Health system interventions to improve the delivery and use of clinical and other preventive services

- Develop quality improvement processes in health systems.
- Promote the use of team-based care in health systems.

Community clinical linkages to support cardiovascular disease and diabetes prevention and control efforts

- Promote the use of diabetes self-management education programs in community settings.
- Promote the use of CDC-recognized lifestyle change programs in community settings for primary prevention of type 2 diabetes.
- Promote the use of non-medical doctor health care providers in the community to support self-management of high blood pressure and diabetes.
- Promote the use of chronic disease self-management programs in community settings.
- Develop policies, processes, and protocols in schools to meet the management care needs of students with chronic conditions.

## Evaluation Approach

CDC is evaluating both the processes and outcomes of State Public Health Actions to document efficiencies and improve programs, expand practice-based evidence, and demonstrate health outcomes. The answers to 4 broad evaluation questions will inform future collaborative efforts. An article on the evaluation approach CDC is using for State Public Health Actions appears in this week’s *Preventing Chronic Disease* (6). Additionally, each state is conducting an evaluation of its own efforts over the 5-year project period, in collaboration with CDC.

## Administration and Management

CDC established an innovative organizational structure to support the administration and management of State Public Health Actions that is designed to facilitate program coordination and collaboration. The functional areas that had previously been performed independently by the individual divisions are now accomplished collectively. For example, previously a program would provide evaluation technical assistance based on the expertise and resources available within the program. Under State Public Health Actions, a group of evaluators representing all of the programs oversees the evaluation of the consolidated program as well as the technical assistance provided to the grantees. Seven workgroups are organized by functions (ie, fiscal management, evaluation support and guidance, program administration and technical assistance, training and conference planning, policy/communication support, epidemiology/surveillance technical assistance, and translation/dissemination guidance). Additionally, integrated teams of project officers and evaluators have been organized into 6 geographical regions consisting of 7 to 11 states each. Leadership for State Public Health Actions is provided by the 4 branch chiefs from the 4 divisions (Heart Disease and Stroke Prevention; Diabetes Translation; Nutrition, Physical Activity, and Obesity; and School Health).

Diversity of expertise within the teams provides opportunities to learn from each other, and the team structure encourages a stronger relationship between program and evaluation, thus elevating the role of evaluation within the grantees’ activities. CDC developed a framework for governance and standard processes for program management that has improved information sharing within and across workgroups and regional teams.

## Technical Assistance and Evaluation Support

Teams of project officers representing each division provide guidance and support to states that are merged into geographic regions. The teams represent all 4 divisions, and each has 4 project officers, 4 evaluators, and a team lead from one of the 4 divisions. Project officers provide technical assistance and arrange for subject matter expertise from both CDC and non-CDC sources to support states’ efforts. Evaluators assist states in developing and executing evaluation plans and provide additional technical assistance as needed. Team members adhere to standard operating procedures to ensure consistency across programs. Regular team meetings promote information sharing and problem solving and provide a forum to address grantee challenges, needs, and successes.

Each team has a lead project officer; lead project officers provide overall coordination for their assigned states. The team leads, project officers, and evaluators meet quarterly for training and to address identified needs.

## Discussion

The framework for State Public Health Actions builds on previous efforts in NCCDPHP to foster coordination and collaboration among programs. This approach, coordinated strategic activities aligned with the 4 domains of public health action, may result in greater program coordination among state and local health, community, and education partners. However, previous findings from similar efforts suggest that coordination and collaboration can be time-consuming, because joint decision making involves communication and negotiation. In addition, the State Public Health Actions program includes various funding streams and reporting requirements that place additional burden on program partners (7). Yet, the benefits resulting from reduced duplication and the potential for improved health outcomes could outweigh the perceived challenges (8). Sharing of evidenced-based best practices across programs, integration of performance measures, and development of new tools and resources may lead to improved health outcomes (9). Furthermore, working within the framework of the 4 domains provides the opportunity to address risk factors and diseases across various settings, including health care, education, and communities, by using multiple population-based approaches (9).

The management of State Public Health Actions across 4 programs has been both challenging and rewarding. The original intent of the approach, to enhance efficiency and impact, has not been completely fulfilled. Although the framework of the 4 domains is clear, implementation of strategies across the domains to facilitate increased synergy among the programs has been a challenge. Other challenges reported by states include hiring restrictions, staff turnover, and time required to process contracts. Additional administrative challenges, such as complex reporting requirements related to fiscal tracking and management systems, have inhibited the exchange of information between the states and CDC. Finally, CDC funding priorities and strategies do not always directly align with those of the states, making program management challenging.

Despite the challenges associated with a collaboration of this magnitude, understanding of each program’s strategies to manage and control diabetes, heart disease, and obesity and to promote school health has increased among those involved with this effort. The structure for managing the program across the 4 categorical areas, while complex, has its strengths. The development of new systems, such as a performance-monitoring database, and processes to facilitate communication, such as peer learning networks, appears to be of benefit both to the grantees and to CDC on the basis of anecdotal feedback from grantees and CDC staff. The coordination among the 4 divisions in delivering technical assistance and training to states may be a model worthy of replication.

Although it is too early to tell whether this program effort will produce the intended outcomes, the results of the evaluation will inform future efforts and point to opportunities for improvement. Considering CDC’s and states’ evolving priorities and the changes in the health care system, implementing evidence-based public health programs to prevent and control chronic diseases is both an opportunity and a challenge.
